# Reevaluation of *Parasynechococcus*-like Strains and Genomic Analysis of Their Microsatellites and Compound Microsatellites

**DOI:** 10.3390/plants11081060

**Published:** 2022-04-13

**Authors:** Jie Tang, Dan Yao, Huizhen Zhou, Lianming Du, Maurycy Daroch

**Affiliations:** 1 School of Food and Bioengineering, Chengdu University, Chengdu 610106, China; tangjie@cdu.edu.cn (J.T.); yaodancdu1113@163.com (D.Y.); zhouhuizhencdu@163.com (H.Z.); dulianming@cdu.edu.cn (L.D.); 2 School of Environment and Energy, Peking University Shenzhen Graduate School, 2199 Lishui Road, Shenzhen 518055, China

**Keywords:** *Parasynechococcus*, microsatellites, SSR, compound microsatellites, genetic diversity

## Abstract

Morphologically similar to *Synechococcus*, a large number of *Parasynechococcus* strains were misclassified, resulting in extreme underestimation of their genetic diversity. In this study, 80 *Synechococcus*-like strains were reevaluated using a combination of 16S rRNA phylogeny and genomic approach, identifying 54 strains as *Parasynechococcus*-like strains and showing considerably intragenus genetic divergence among the subclades identified. Further, bioinformatics analysis disclosed diversified patterns of distribution, abundance, density, and diversity of microsatellites (SSRs) and compound microsatellites (CSSRs) in genomes of these *Parasynechococcus*-like strains. Variations of SSRs and CSSRs were observed amongst phylotypes and subclades. Both SSRs and CSSRs were in particular unequally distributed among genomes. Dinucleotide SSRs were the most widespread, while the genomes showed two patterns in the second most abundant repeat type (mononucleotide or trinucleotide SSRs). Both SSRs and CSSRs were predominantly observed in coding regions. These two types of microsatellites showed positive correlation with genome size (*p* < 0.01) but negative correlation with GC content (*p* < 0.05). Additionally, the motif (A)_n_, (AG)_n_ and (AGC)_n_ was a major one in the corresponding category. Meanwhile, distinctive motifs of CSSRs were found in 39 genomes. This study characterizes SSRs and CSSRs in genomes of *Parasynechococcus*-like strains and will be useful as a prerequisite for future studies regarding their distribution, function, and evolution. Moreover, the identified SSRs may facilitate fast acclimation of *Parasynechococcus*-like strains to fluctuating environments and contribute to the extensive distribution of *Parasynechococcus* species in global marine environments.

## 1. Introduction

Genus *Synechococcus* from the family Synechococcaceae and *Prochlorococcus* from the family Prochlorococcaceae are the dominant members of the picophytoplankton and are considered major contributors to the global carbon cycle [1]. Of the two, *Synechococcus* represents a polyphyletic group that comprises strains originating from freshwater, seawater, and brackish water [2]. *Synechococcus* has been a cryptic genera due to difficulties in differentiation based on morphology [3]. Recent studies indicated that *Synechococcus* strains from marine and brackish water were distantly related to freshwater *Synechococcus* strains as suggested by molecular data. They were proposed to be a new genus into the family Prochlorococcaceae, *Parasynechococcus* [3,4]. Within the family Prochlorococcaceae, *Parasynechococcus* was phylogenetically a sister clade to marine *Prochlorococcus*, which were both genetically related to the freshwater *Cyanobium gracile* [5]. To date, only a small population of *Synechococcus* strains was taxonomically revised to *Parasynechococcus* [6], and numerous *Synechococcus*-like strains were unclassified into the actual taxonomy. Therefore, the diversity of *Parasynechococcus* might be severely underestimated and is expected to be explored by undergoing an extensive revision on the taxonomy of *Synechococcus*-like strains. Moreover, as *Parasynechococcus* play a focal role in the microbial food web in marine environments, it is very interesting to establish their phylogenetic relationships and evolutionary changes in their genomes.

Meanwhile, microsatellites, also named simple sequence repeats (SSRs), typically comprise tandemly repeated units in genomes [7]. SSRs were usually 1–6 bp in length and distributed across the genome, including coding and noncoding regions [8]. The high abundance and length hypervariability of SSRs have been widely observed in Cyanobacteria [9,10]. These characteristics of SSRs are important for the determination and understanding of cyanobacterial genome evolution by investigating the differences in SSRs length, duplications, replication slippage, point mutations, and DNA repair across the entire genomes [11]. Moreover, a previous study indicated that the instability of SSRs could be utilized by prokaryotes as potential drivers for genetic plasticity and prokaryotic adaptation on short-term evolutionary time scales, without contributing significantly to the overall accumulation of mutations [12]. Furthermore, researchers are interested in SSRs due to their considerable applications in DNA fingerprinting, genetic mapping, gene regulation, population genetics, and evolution [7,13,14]. Additional to SSRs, compound microsatellites (CSSRs) are defined as comprising of at least two individual SSRs, e.g., (AGC)_n_-(AG)_n_-(G)_n_. CSSRs are more polymorphic than their single counterparts [15]. Various evolutionary and genomic features of CSSRs were previously studied in other microorganisms. Notably a recent on Cyanobacteria, e.g., *Leptolyngbya* [9] described the distribution and diversity of microsatellites among these filamentous strains.

Recently, computational mining from genome sequences has gradually replaced traditional SSR mining grounded on genomic libraries [16]. Next-generation sequencing (NGS) has been extensively employed to investigate cyanobacterial genomes [17,18,19]. This offers an opportunity to explore cyanobacterial SSRs and CSSRs on a genome-wide scale and to resolve the relationship of closely related species at the genomic level in addition to the phylogeny traditionally based on 16S rRNA. Moreover, the characterization of SSRs and CSSRs is a prerequisite for future studies regarding their distribution, function, and evolution.

To date, there is only one genome available for *Parasynechococcus* based on the record of the National Center for Biotechnology Information (NCBI). Nevertheless, this was probably ascribed to the fact that numerous strains were misclassified as *Synechococcus* in light of the highly similar morphology between *Synechococcus* and *Parasynechococcus* [20]. Thus, reevaluation of these *Synechococcus* strains on NCBI is essential for diversifying the genus *Parasynechococcus* and for providing a better resolution of genetic diversity among these *Parasynechococcus*-like strains. In the current study, we resolved the relationship between *Synechococcus*-like strains and *Parasynechococcus* strains using combined 16S rRNA and genome-based approach. Further, the genomes of *Parasynechococcus*-like strains were mined and analyzed for SSRs and CSSRs to elucidate their patterns of distribution, abundance, density, and diversity. The study offers the first insight into SSRs and CSSRs in *Parasynechococcus* genomes and might be beneficial for functional and evolutionary studies of these simple sequence repeats identified in *Parasynechococcus* strains.

## 2. Results

### 2.1. Reevaluation of Synechococcus-like Strains

**Figure 1:**
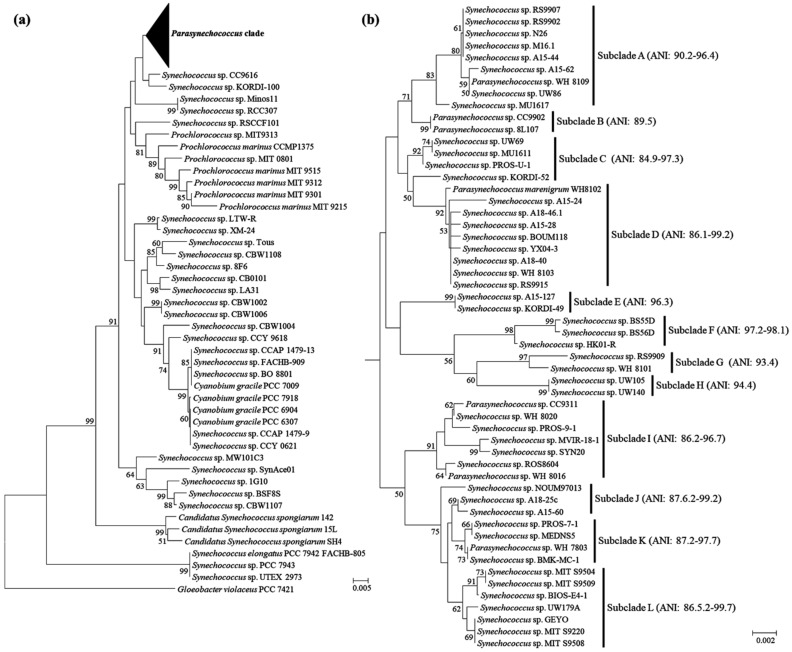
Phylogenetic tree (**a**) of 16S rRNA gene sequences. *Parasynechococcus* clade was shown in subtree (**b**). The ANI values among strains within subclades were shown in brackets. The scale bar indicated substitutions per site.

After filtration using the criteria described in the method, we constructed a high-quality dataset comprising a total of 80 *Synechococcus*-like strains, with genome completeness >91% (completeness of 76 genomes >98.9%) and contamination <2% (Table S1). The 80 *Synechococcus*-like strains, together with cyanobacterial reference strains, were subjected to the phylogenetic inference of their 16S rRNA sequences. As expected, these *Synechococcus*-like strains were phylogenetically classified into different clades (Figure 1a). Nevertheless, none of the surveyed *Synechococcus*-like strains was phylogenetically categorized into the genus *Synechococcus* [21], reinforcing the necessity of reevaluation of these *Synechococcus*-like strains. The majority (54 strains) of *Synechococcus*-like strains clustered in a clade (Figure 1b), including seven strains previously identified as *Parasynechococcus* based on molecular data and morphological characterization [3]. Five *Synechococcus*-like strains (Figure 1a) were closely related to the genus *Cyanobium* [5]. The remaining 21 strains were scattered across the 16S phylogram and appeared to be phylogenetically novel to the described taxa (Figure 1a), suggesting the presence of underlying new genera. The genome-wide ANI (average nucleotide identity) analysis further verified the phylogram-based speculation. Compared to the previously established taxon (*P. marenigrum* WH8102, *P. marinus* MIT 9313, *C. gracile* PCC 6307), 20 *Synechococcus*-like strains showed ANI values below 81% (Table S2). Only *Synechococcus* sp. CCY 9618 exhibited an ANI value of 84.6% with *C. gracile* PCC 6307 (Table S2). According to the suggested values for genus (ANI < 83%) delimitation [22,23], the ANI results herein confirmed the presence of new genera among these *Synechococcus*-like strains.

Remarkably, the 54 *Parasynechococcus*-like strains appear to be further divided into 12 subclades in the 16S phylogram (Figure 1b), most of which were supported by high bootstrap values. To further resolve the relationship of *Parasynechococcus*-like strains, ANI values were calculated based on their genomes. Within each subclade in the 16S phylogram, the genome-wide ANI values among strains were all higher than 84%, varying from 84.9 to 99.2% (Figure 1b). Pairwise comparisons among genomes of representative strains from each subclade indicated that the ANI values of all the pairwise comparisons ranged from 77.5 to 80.5% (Table 1). Based on the suggested cut-off values for genus (ANI < 83%) and species (ANI > 96%) delimitation [22,23], the results of genome-wide ANI analysis not only verified the classification of subclades based on the 16S phylogram, but also showed the genomic divergence among these *Parasynechococcus* subclades. However, the identification of *Parasynechococcus*-like strains herein may be problematic as only genetic data were used without sufficient morphological characterization. Based on both the bacteriological and botanical codes [24,25], delimitation of novel cyanobacteria or taxonomic reclassification usually requires a combination of phylogenetic, cytomorphological and ecological markers [18,19,26,27]. Therefore, future studies are essential to confirm and verify these *Parasynechococcus*-like strains.

### 2.2. Number, Relative Abundance (RA), and Relative Density (RD) of SSRs and CSSRs

**Figure 2:**
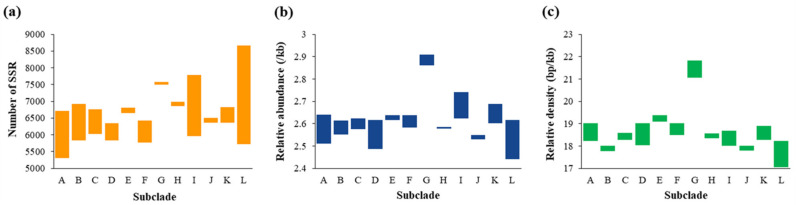
Number (**a**), relative abundance (**b**), and relative density (**c**) of SSRs in genomes of 54 *Parasynechococcus*-like strains. Data were presented by the subclades (A–L) identified in Figure 1b.

Based on the results of molecular identification, 54 *Parasynechococcus*-like strains were further compiled for subsequent analysis. Throughout the analysis, the total number of 352,765 SSRs were identified and disproportionally distributed across the genomes of 54 *Parasynechococcus*-like strains (Table S3). The number of SSR (nSSR) in each of the surveyed genomes varied from 5303 (*Synechococcus* sp. M16.1) to 8668 (*Synechococcus* sp. BIOS-E4-1). Uneven distributions of SSRs were presented among subclades (Figure 2a). A varying RA and RD of SSRs were also observed (Table S3), ranging from 2.44/kb (*Synechococcus* sp. GEYO) to 2.91/kb (*Synechococcus* sp. RS9909) and from 17.06 bp/kb (*S. elongatus* GEYO) to 21.82 bp/kb (*Synechococcus* sp. RS9909), respectively. At the subclade level, the RA (2.86–2.91/kb) and RD (21.06–21.82 bp/kb) values of subclade G were significantly higher than that of other subclades (Figure 2b,c), whereas the RA and RD values of the other 11 subclades fluctuated around 2.60/kb and 18.40 bp/kb, respectively.

**Figure 3:**
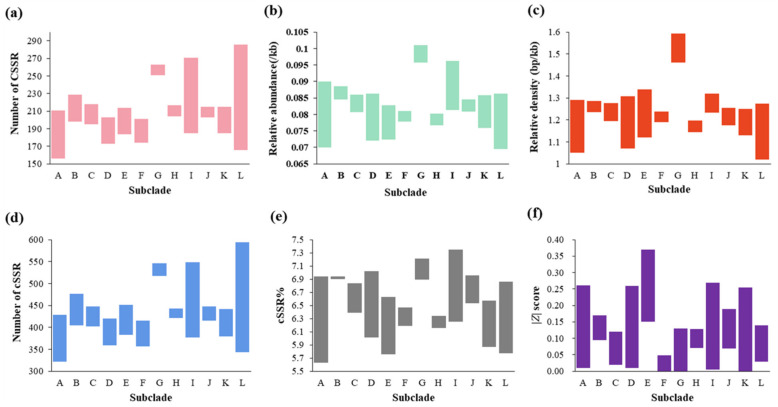
Number (**a**), relative abundance (**b**), and relative density (**c**) of CSSRs in genomes of 54 *Parasynechococcus*-like strains; (**d**) number of cSSR in each genome; (**e**) cSSR% = ncSSR/nSSR, percentage of individual SSRs being part of CSSRs; (**f**) |Z|, statistical significance of CSSR representation. Data were presented by the subclades (A–L) identified in Figure 1b.

Meanwhile, the results indicated that a total of 11,112 cSSRs (an individual SSR being a component of a CSSR) were identified in the genomes of 54 *Parasynechococcus*-like strains (Table S3). The number of CSSR (nCSSR) in each genome greatly varied from 156 (*Synechococcus* sp. UW86) to 286 (*Synechococcus* sp. BIOS-E4-1), and nCSSR tremendously shifted among subclades (Figure 3a). Considerable variations were shown by RA and RD of CSSRs, fluctuating from 0.07 to 0.10/kb and from 1.02 to 1.59 bp/kb, respectively (Table S3). Analogously, subclade G showed higher RA and RD of CSSRs than the other subclades (Figure 3b,c). Variations of RA and RD of CSSRs were evident among subclades, while limited variations were exhibited within the subclade.

The number of cSSR (ncSSR) among genomes shifted from 322 to 594 (Figure 3d, Table S3). It was suggested that less than 8% of all SSRs in each genome had a compound motif as revealed by cSSR% (Figure 3e). Subclades A, D, E, I, K, and L showed a wider range of cSSR% variation compared to the other subclades. Although strains shared similar RA and RD of SSR and CSSR within subclades or from different subclades, the variations in cSSR% suggested that the proportion of SSRs engaging in CSSR was discordant among strains (Table S3). The Z scores revealed that the representation of CSSR was statistically significant. The nCSSR_obs_ was less than nCSSR_exp_ in 27 genomes (Table S3). The remaining 27 genomes, on the contrary, showed more nCSSR_obs_ than nCSSR_exp_. The genome of *Synechococcus* sp. KORDI-49 exhibited the greatest statistical significance. Strains only within subclades D, E, F, and K showed a consistent pattern of Z score, positive or negative value, while strains within the other subclades exhibited both types of values.

### 2.3. Distribution and Diversity of SSRs

**Figure 4:**
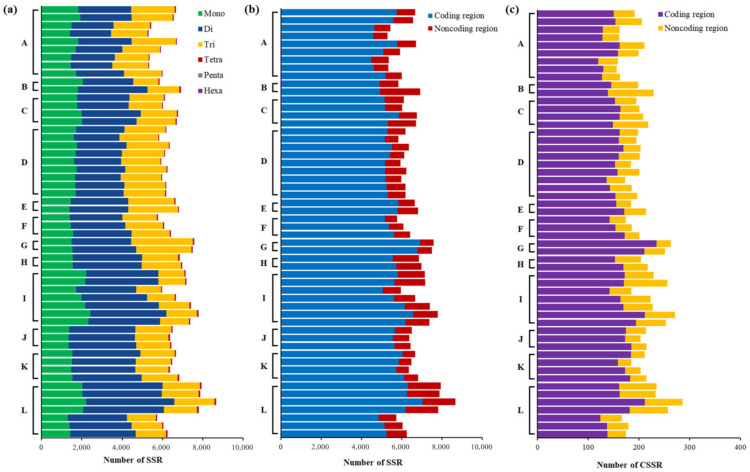
The SSR and CSSR distribution patterns of 54 *Parasynechococcus*-like strains genomes: (**a**) distribution according to SSR repeat type; (**b**) SSR distribution according to coding and non-coding regions; and (**c**) CSSR distribution according to coding and non-coding regions. Data were presented by the subclades (A–L) identified in Figure 1b.

Mono-, di-, and tri- nucleotides were obligated to the vast majority (98.9–99.6%) of SSRs in each genome (Figure 4a). Dinucleotide repeats were the most abundant SSR type (37.4–51.9%) in all genomes except for the genome of *Synechococcus* sp. RS9909, in which the number of trinucleotide (3053) was marginally higher than that of dinucleotide (2954). Excluding the case of RS9909, 36 genomes followed the pattern: di- > tri- > mononucleotide SSRs, while 17 genomes followed the pattern: di- > mono- > trinucleotide SSRs. A consistent pattern was observed within all the subclades except for subclade L, which contained both patterns.

SSRs were overwhelmingly located in coding regions of all the 54 genomes analyzed (Figure 4b), accounting for 71.3–91.4% of SSRs. Only low percentages of SSRs (8.6–28.7%) were found to be distributed in noncoding regions. Moreover, very limited variations in the percentage of SSRs in coding/noncoding regions were noticed within subclades except for subclade B. Within subclade B, the percentage of SSRs in coding regions was 83.7% in *Parasynechococcus* sp. CC9902, while 71.3% in *Parasynechococcus* sp. BL107. The variations within or between subclades indicated that the degree of SSRs potentially involved in the function and evolution of coding genes was distinguishable among strains.

**Figure 5:**
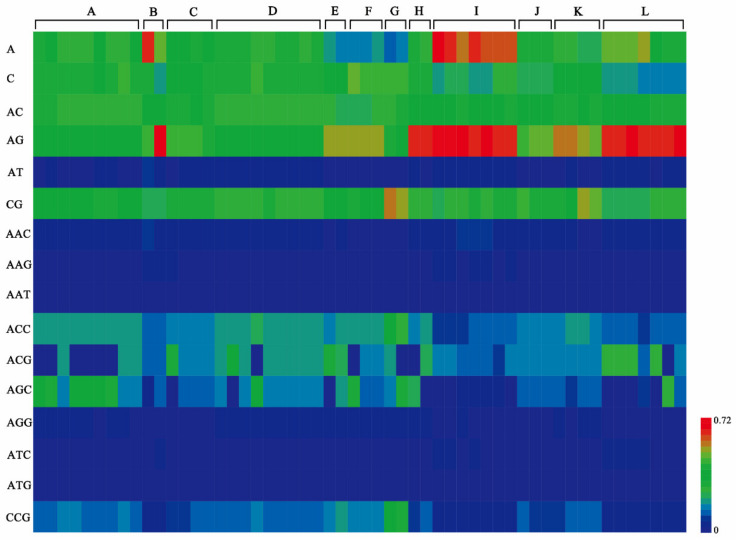
Relative abundance of standard motifs in mononucleotide, dinucleotide, and trinucleotide repeat type identified in genomes of 54 *Parasynechococcus*-like strains. The relative abundance of each motif was represented by a color block, whose color was proportional to the values (/kb) as indicated by the scale bar. Each vertical bar represented the motifs from one genome, and genomes were grouped by the subclades (A–L) identified in Figure 1b. Heatmap was generated using the gplots package in R v3.6.2.

Moreover, we constructed a heatmap (Figure S1) to present the RA of the identified 239 standard motifs in each genome. Clear discrepancies among genomes within subclades or beyond subclades were mainly observed in the RA of motifs in mononucleotide (0.13–0.65/kb), dinucleotide (0.01–0.72/kb), and trinucleotide (0.01–0.42/kb) repeat type (Figure 5). The motif (A/C)_n_ was the only two mononucleotide repeat type in each genome. Among the four dinucleotide SSRs motif, the most abundant dinucleotide repeat types were (AG)_n_, (AC)_n_, and (CG)_n_. Among the trinucleotide repeat type, the most abundant motifs were (AGC)_n_, (ACG)_n_, (ACC)_n_, and (CCG)_n_. The RA of tetranucleotide, pentanucleotide, and hexanucleotide SSR motifs were almost identical among genomes (Figure S1).

### 2.4. Complexity, Motifs, and Distribution of CSSRs

CSSR comprises individual SSR with different numbers, leading to different complexity (C: the number of cSSRs in a CSSR). On the whole, the complexity of CSSRs in the 54 genomes varied from 2 to 9 (Table S4), but complexity = 2 accounted for 94.9%, followed by complexity = 3 (4.6%). A very small part (0.5%) of CSSRs showed complexity ≥4. Of the CSSRs, only four CSSRs exhibited high complexity (≥6). It was observed that the number of CSSR was reversibly correlated to its complexity. Limited variations were observed within subclades or among subclades due to complexity = 2 or 3 in the vast majority of CSSRs.

Unique motifs were found in all the 54 genomes (Table S5). Their number immensely fluctuated amongst genomes, from 2 to 42. Extensive variations of unique motif numbers also existed within subclades. On the basis of these data, the significant diversity of motifs among the genomes can be concluded.

CSSRs, consistent with SSRs, were also overwhelmingly located in coding regions of all the 54 analyzed genomes (Figure 4c), ranging from 60.7 to 89.4%. The distribution pattern was in line with the common evidence that SSRs and CSSRs in prokaryotic genomes occurred predominantly in their coding regions [9,28].

### 2.5. Pearson Linear Correlation

Four factors that may be related to SSR and CSSR occurrence were statistically analyzed, and results were summarized in Table 2. The nSSR showed significantly positive correlation with genome size (ρ = 0.96, *p* < 0.01) and negatively correlation with GC content (ρ = −0.40, *p* < 0.01). The nCSSR was significantly positively correlated with genome size (ρ = 0.82, *p* < 0.01), nSSR (ρ = 0.91, *p* < 0.01), and ncSSR (ρ = 1.00, *p* < 0.01), while having significantly negative correlation with GC content (ρ = −0.40, *p* < 0.01). To summarize, the degree of correlation with nCSSR was in the following order ncSSR > nSSR > genome size > GC content.

## 3. Discussion

The taxonomic classification of *Synechococcus*-like cyanobacteria has been controversial due to their simple unicellular morphology. The polyphyly and phylogenetic diversity of *Synechococcus*-like species revealed by molecular analysis [29,30] emphasized the necessity of their accurate taxonomic reclassification. This will facilitate the establishment and diversity expansion of novel taxon. Consequently, several *Synechococcus*-like species have been proposed as new taxon [3,23,31]. More recently, the genus *Parasynechococcus*, morphologically similar to *Synechococcus*, has been validly described [3]. However, the diversity of *Parasynechococcus* was extremely underestimated according to the record in the public database. In this study, the phylogeny of 16S rRNA and genomic approach were used to reevaluate 80 *Synechococcus*-like strains. A total of 54 strains was identified as *Parasynechococcus*-like strains as suggested by the 16S phylogram (Figure 1). This was a great supplement to the *Parasynechococcus* diversity as shown in a previous study (seven *Parasynechococcus* strains) [3] or NCBI record (only one *Parasynechococcus* strain). Moreover, the ecological data of these *Parasynechococcus*-like strains all implied marine origins as indicated by the isolation source (Table S3). This strongly supported and verified the separation of *Parasynechococcus* from *Synechococcus*, the species which originated from a freshwater habitat [32]. Although genomic analysis indicated considerably genetic divergence (Table 1) among the subclades identified in the 16S phylogram (Figure 1b), very limited information (e.g., morphology) on most *Parasynechococcus*-like strains was available, hindering comprehensive investigation of accurate taxonomy. Therefore, more studies are necessary for the future since polyphasic evaluation is a standard procedure for taxonomic allocation of cyanobacteria.

Focusing on *Parasynechococcus*-like strains, we have analyzed the following parameters of SSRs and CSSRs in 54 genomes: occurrence, abundance, and composition. Dissimilarity patterns of SSRs distribution among these genomes were revealed by the results (Figure 2, Table S3), indicating that SSRs might be one of the contributors to the genetic diversity of *Parasynechococcus* genomes and may suggest that rapid evolutionary changes are underway in these genomes [33]. Contrary to the dissimilarity patterns among genomes from different subclades, a high degree of a consistent pattern of SSRs distribution was exhibited by genomes within the subclade. Therefore, the genomic discrepancy among subclades, as suggested by ANI analysis (Table 1), might lead to the dissimilarity patterns of SSRs distribution. Dinucleotides were the most abundant repeat type in genomes of 53 *Parasynechococcus*-like strains. The results were consistent with the dominance of mononucleotide or dinucleotide repeats in prokaryotic genomes [9,34], whereas mononucleotide repeats were typically dominant SSRs identified in eukaryotic genomes, e.g., *Agaricus bisporus* [35] and human chromosomes [36]. In addition, the two distribution patterns of SSR repeat type observed (Figure 4a) could indicate structural dissimilarity in genomes between subclades.

The GC content ranged from 52.45% to 64.44% across the genomes of 54 *Parasynechococcus*-like strains (Table S1). Intriguingly, the negative correlation of GC content was observed with nSSR and nCSSR (ρ = −0.40/−0.40, *p* < 0.01). The GC content of the genome might affect the GC content of SSRs, further influencing the marker developments in light of the difficult amplification of GC-rich SSRs by PCR. Taken together, the AT-rich SSRs identified in this study (Figure S1) may be beneficial for future exploitation of SSRs markers. The genome sizes of the 54 genomes ranged from 1.68 Mb to 3.31 Mb (Table S1). Though smaller genomes possessing more SSRs or CSSRs were observed, e.g., *Synechococcus* sp. PROS-7-1 (Tables S1 and S3), the Person analysis indicated a positively association between genome size and nSSR/nCSSR (ρ = 0.96/0.22, *p* < 0.01) (Table 2). Though the SSR number of the genomes of 54 *Parasynechococcus*-like strains (5303 to 8668) was comparable to that of other cyanobacterial genomes (2283 to 53,041) deposited in the public microsatellite database (http://big.cdu.edu.cn/psmd/, (accessed on 1 January 2022)), evident distinctions of SSR number existed at the genus level, e.g., *Leptolyngbya* strains (11,086 to 24,000), *Thermosynechococcus* (7490 to 7724) and *Tolypothrix* (32,706 to 37,800). Noticeably, these data confirmed the result of the correlation between nSSR/nCSSR and genome size (Table 2).

Variations in cSSR% were also noticed between *Parasynechococcus* genomes and other cyanobacterial genomes. In the genomes of 54 *Parasynechococcus*-like strains, cSSR% ranged from 5.63% to 7.35% (Table S3), suggesting a low percentage of SSRs participating in the formation of CSSRs. This range was within cSSR% span (4.52–19.44%) of cyanobacterial genomes based on the public microsatellite database (http://big.cdu.edu.cn/psmd/, (accessed on 1 January 2022)). Expectedly, various cSSR% were obtained in different organisms, e.g., 8.48–9.29% in *Gloeobacter* genomes, 28.79% in rat genome, and 32.79% in the human papillomavirus genome [34]. These results indicated that SSR and CSSRs may play different roles in genetic polymorphism among organisms.

The CSSRs identified in the genomes of 54 *Parasynechococcus*-like strains were dominantly composed of two SSRs (complexity = 2) (Table S4). The number of CSSRs was reversibly correlated to their complexity. The motifs in CSSRs differed among the surveyed genome (Table S4). A total of 941 unique motifs were detected in the 54 genomes (Table S5). Two possible causes formed these unique motifs. The SSR types were different in each genome, further leading to miscellaneous SSR-couple. Conversely, the same SSR type might contain distinct nucleotides. Many unique motifs could be distinguished from one another by a single or a couple of mutations, which probably contributed by frequent mutation rates and evolutionary processes in each organism [37].

Given the more compact genomes of cyanobacteria to those of eukaryotes, SSRs and CSSRs identified in this study, as expected, both occurred predominantly in coding regions of each genome (Figure 4b,c). The distribution of SSRs and CSSRs in coding regions suggested a potential functional role in affecting genome organization, gene regulation, transcription, and protein function [7,8]. In particular, SSRs located in coding regions are typically subject to stronger selective pressure than those in non-coding regions due to their functional necessity [38]. The selection pressure may give rise to systematic directional mutations of the repeat sequences that eventually bring about the change of function and/or expression levels of the adaptation-relevant genes and relaxation of the stress [39]. Herein, massive SSRs were found in each genome of 54 *Parasynechococcus*-like strains (Table S3). These SSRs may facilitate fast acclimation of *Parasynechococcus*-like strains to fluctuating environments and may play a crucial part in the cosmopolitan distribution of *Parasynechococcus*-like strains to universally marine niches (Table S3). The varying compositions of SSRs distributed in coding regions among phylotypes or subclades might imply a diverse degree of participation in evolution or functions. Nevertheless, the underlying functions, together with the mutation mechanism, remained largely unclear [40]. Currently, SSR variation was prevailingly ascribed to replication slippage and recombination [41]. Furthermore, the extensive presence of overlapping genes in bacterial genomes possibly brought about additional impacts induced by SSRs or CSSRs. It would be very interesting for future studies to illustrate the evolutionary part of SSRs and their impact on how organisms cope with various environmental stresses. It must be aware that horizontal gene transfer (HGT) is a non-negligible force of genetic diversification and the evolution of prokaryotes [42].

*Parasynechococcus*-like strains showed extensively genetic diversity as revealed by 16S phylogram and genomic analysis. Further, disclosure of the patterns regarding distribution, abundance, diversity, and density of both SSRs and CSSRs in genomes of *Parasynechococcus*-like strains is essentially a prerequisite prior to demonstrating their functional and evolutionary role. The variations observed in this study confirmed the contributing role of SSRs in genome polymorphism [12]. Moreover, the SSR variability might be thought of as the most important driver of genomic plasticity, hence allowing targeted mutation and evolution. The SSRs identified may facilitate fast acclimation of *Parasynechococcus*-like strains to fluctuating environments and may play a vital role in the extensive distribution of *Parasynechococcus* species to global marine niches. Overall, this study laid a solid basis for an understanding of the genomic distribution, functions, and evolution of microsatellites and compound microsatellites in *Parasynechococcus* genomes.

## 4. Materials and Methods

### 4.1. Genome Sequences

From the genomic resources of the NCBI at the time of this study (2021/12/1), all *Parasynechococcus* genomes were retrieved as a preliminary dataset. *Synechococcus* genomes in the NCBI were also downloaded to complement the dataset. Filtrations were performed to ensure the focus of *Parasynechococcus*-like strains. First, *Synechococcus* strains that were proposed to be non-*Parasynechococcus* genus were removed by conducting literature searches, e.g., strain JA-2-3Ba and JA-3-3Ab reclassified into a newly delineated taxon, genus *Thermostichus* [3]. Second, to avoid data redundancy and biased genome representation, the remaining dataset was subjected to the calculation of whole-genome average nucleotide identity (ANI) for pairwise genome comparisons using the ANI calculator with default settings (http://enve-omics.ce.gatech.edu/ani/, (accessed on 15 December 2021). Genomes with an ANI value greater than 99.9% were considered redundant genomes, and then only one of these genomes was randomly kept. Third, the quality of the remaining genomes was evaluated using CheckM [43] to ensure a more reliable genome dataset with near completeness (≥90%) and low contamination (<5%). To illustrate the relationship among the remaining strains, the 16S rRNA gene was extracted from the genomes for phylogenetic analysis. The genomes assembled at the level of scaffold or contig were susceptible to the case that no or short 16S rRNA sequences were available. Thus, strains with such cases were also removed from the dataset. Finally, all the filtration generated a dataset comprising 80 *Synechococcus*-like strains. Detailed genome information regarding these strains was summarized in Table S1.

### 4.2. Phylogenetic Analysis and Genome Comparison

Sequences of the 16S rRNA gene were collected for the 80 *Synechococcus*-like strains by extraction from the genome sequences and for cyanobacterial references through BLAST search. Sequences were aligned, trimmed, and edited in Mega7 [44], generating an alignment with a length of 1284 bp. Phylogenetic analyses were performed as described [45,46]. Briefly, phylogenetic analyses were conducted using PhyML v3.0 [47], and the substitution models were selected by the Model Selection function implemented in PhyML [48] under Akaike information criterion (AIC). A non-parametric bootstrap test (1000 replications) was performed to assess the robustness of tree topologies. ANI analysis was performed for genome comparison as described above.

### 4.3. Detection and Analysis of SSRs and CSSRs

Through the aforementioned analysis, 54 *Parasynechococcus*-like strains were identified, and their genome sequences and genomic annotations were used for subsequent analysis. The individual SSRs and CSSRs were detected in each genome using repeat search engine Krait v1.2.2 [49]. Upon small genomes in *Parasynechococcus*-like strains, the minimum repeats for mono-, di-, tri-, tetra-, penta-, and hexanucleotide microsatellites were customized to 6, 3, 3, 3, 3, and 3, respectively. The maximum distance (*d*max = 10 bp) between any two adjacent SSRs was selected to analyze the existence of their compound variants. All the aforementioned parameters in the study were set based on the empirical criterion of previous studies on prokaryotic genomes [9,15,49]. Krait was used to map and correlate coordinates of all identified SSRs and CSSRs onto coding and noncoding regions of the genomes. The complexity and motifs of CSSRs were analyzed as well.

### 4.4. Statistical Analysis

For convenience, statistical terms used in this study were abbreviated as follows. nSSR: number of SSRs in each genome; nCSSR: number of CSSRs in each genome; cSSR: individual SSR being a component of a CSSR; C: complexity defined by the number of cSSRs in a CSSR; ncSSR: number of cSSR in each genome; and cSSR%: percentage of ncSSR that contribute to nSSR in each genome (cSSR% = ncSSR/nSSR). To alleviate the effect of genome size on the comparative analysis, the numbers of SSRs and CSSRs were normalized as relative abundance (RA), the number of SSRs and CSSRs per kb of the genome sequence studied, and relative density (RD), the total length contributed by each SSRs and CSSRs per kb of the genome sequence studied.

The effects of genome size, GC content, nSSR, nCSSR, and ncSSR were studied using the Pearson correlation coefficient (ρ) and calculated using cor.test function in R v3.6.2. Significance levels of 0.05 and 0.01 were applied for the analysis. *Z* index [50] was used to evaluate the statistical significance of CSSR represented in each genome. The following equations were used to calculate relevant Z scores:

(1) $$\bar{C}=\frac{1}{n}\overset{n}{\underset{i=1}{\sum }}\left(\frac{{\mathrm{ncSSR}}_{i}}{{\mathrm{nCSSR}}_{i}}\right)$$

(2) $${\mathrm{nCSSR}}_{\exp}=\frac{{\mathrm{ncSSR}}_{i}}{\bar{C}}$$

(3) $$Z=\frac{\left({\mathrm{nCSSR}}_{\mathrm{obs}}-{\mathrm{nCSSR}}_{\exp}\right)}{\sqrt{{\mathrm{nCSSR}}_{\exp}}}$$

where n, total number of genomes (n = 54); i, genome order; ${\mathrm{ncSSR}}_{i}$, number of cSSR in genome; nCSSR_i_ (also called nCSSR_obs_), observed number of CSSRs in genome; $\bar{C}$, average of complexity of 54 genomes ($\bar{C}$ = 2.059 in this study); and nCSSR_exp_, expected number of CSSRs in genome.

## 5. Conclusions

In this study, 80 *Synechococcus*-like strains were reevaluated using a combination of 16S rRNA phylogeny and genomic approach, identifying 54 strains as *Parasynechococcus*-like strains. The position of *Parasynechococcus* as a sister clade to *Prochlorococcus* was confirmed within this study, and new characteristics for *Parasynechococcus* were proposed. Further, bioinformatics analysis disclosed diversified patterns of distribution, abundance, density, diversity of microsatellites (SSRs), and compound microsatellites (CSSRs) in genomes of these *Parasynechococcus*-like strains. This study provides the first insight into SSRs and CSSRs in genomes of *Parasynechococcus*-like strains and will be useful as a prerequisite for future studies regarding their distribution, function, and evolution. Moreover, the SSRs identified may facilitate fast acclimation of *Parasynechococcus*-like strains to fluctuating environments and may play a vital role in the extensive distribution of *Parasynechococcus* species to global marine niches.

## Figures and Tables

**Table 1:** ANI values among genomes of representative strains from each *Parasynechococcus* subclade identified in Figure 1b.

Subclade	Strain	M16.1	8L107	PROS-U-1	WH 8102	KORDI-49	BS56D	WH 8101	UW105	MVIR-18-1	A18-25c	WH 7803	BIOS-E4-1
A	M16.1	100											
B	8L107	77.5	100										
C	PROS-U-1	82.0	77.5	100									
D	WH 8102	80.6	77.7	80.2	100								
E	KORDI-49	79.9	78.2	79.6	80.1	100							
F	BS56D	78.9	78.0	79.8	79.0	78.8	100						
G	WH 8101	78.6	77.7	78.6	78.9	78.6	81.7	100					
H	UW105	78.5	77.9	78.7	78.1	78.0	78.2	77.8	100				
I	MVIR-18-1	77.9	78.9	78.2	78.5	77.7	77.8	77.8	77.5	100			
J	A18-25c	80.3	77.9	80.0	79.7	79.0	78.9	78.6	78.3	77.6	100		
K	WH 7803	79.7	77.9	79.7	80.3	79.6	79.1	79.0	78.0	78.0	79.3	100	
L	BIOS-E4-1	78.8	78.2	78.8	78.3	78.9	78.8	78.0	78.3	78.1	78.2	78.0	100

**Table 2:** Pearson analysis between nCSSR and genome size, GC content, nSSR, and ncSSR.

		Genome Size	GC Content	nSSR	ncSSR
nSSR	ρ	0.96	−0.40	-	0.91
	Significance	*p* < 0.01	*p* < 0.01	-	*p* < 0.01
nCSSR	ρ	0.82	−0.40	0.91	1.00
	Significance	*p* < 0.01	*p* < 0.01	*p* < 0.01	*p* < 0.01

## Data Availability

The data presented in this study are openly available in the National Center for Biotechnology Information (https://www.ncbi.nlm.nih.gov/genome/, (accessed on 1 November 2021).

## Supplementary Materials

The following are available online at https://www.mdpi.com/article/10.3390/plants11081060/s1, Figure S1: Relative abundance of standard motifs identified in genomes of 54 *Parasynechococcus*-like strains, Table S1: Detailed genome information regarding 80 *Synechococcus*-like strains used in this study, Table S2: ANI values between *Synechococcus*-like strains and representative strains from family Prochlorococcaceae, Table S3: Summary of SSRs and CSSRs in genomes of 54 *Parasynechococcus*-like strains, Table S4: Detailed information on CSSRs identified in genomes of 54 *Parasynechococcus*-like strains, Table S5: Unique motifs of CSSR identified in each genome.
