# Supplementary material for: Reevaluation of Parasynechococcus-like Strains and Genomic Analysis of Their Microsatellites and Compound Microsatellites
Source: Plants (Basel). 2022 Apr 13;11(8):1060. doi: 10.3390/plants11081060 (PMC9024877; doi:10.3390/plants11081060)
Supplement: Supplementary file 1 [file plants-1638403-Supplementary.pdf › plants-1638403-Supplementary.pdf]

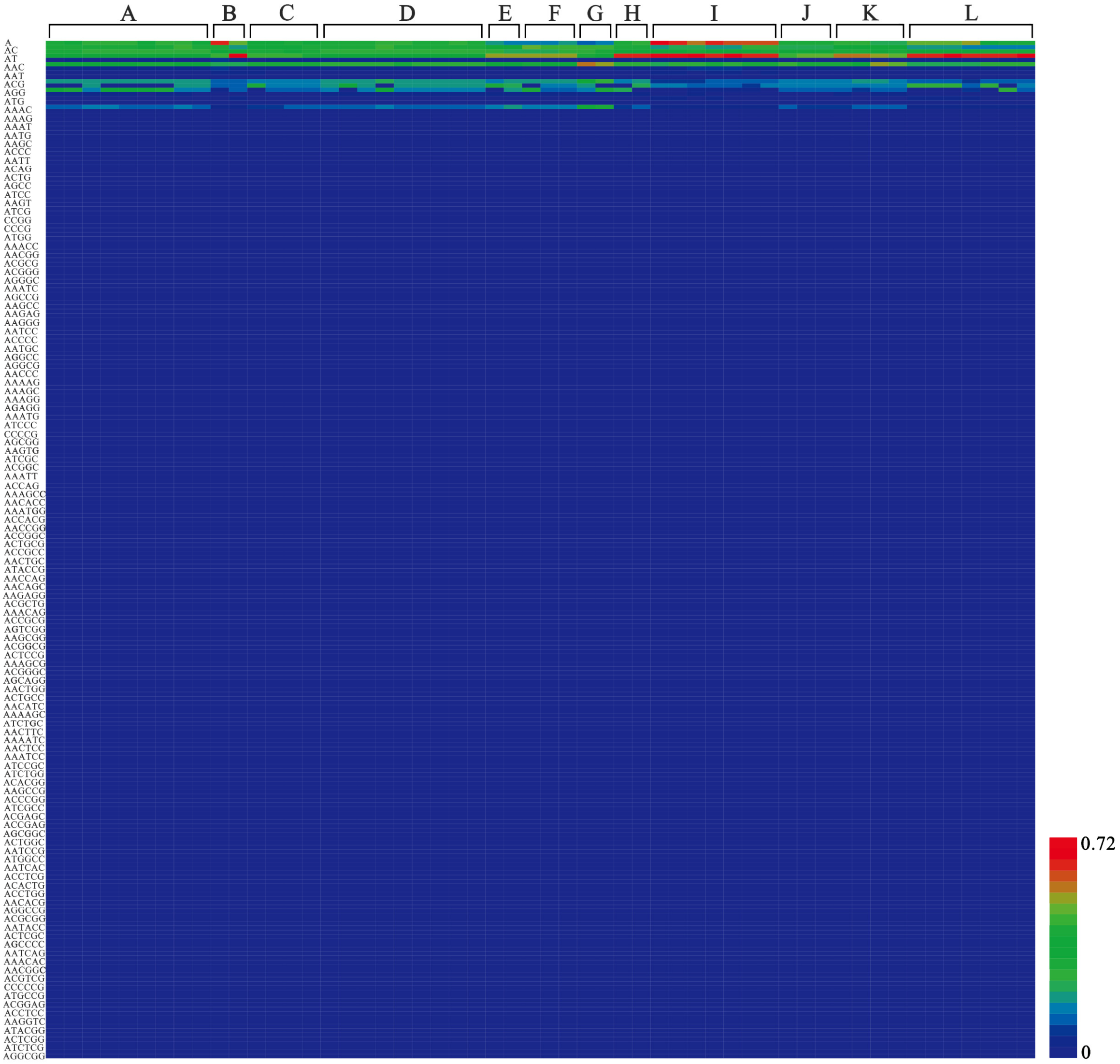

Figure S1. Relative abundance of standard motifs identified in genomes of 54 *Parasynechococcus*-like strains.
